# Usefulness of bilateral cerebral regional oxygen saturation measurements in determining selective cerebral perfusion flow rate in a pediatric patient with aortic arch stenosis: a case report

**DOI:** 10.1186/s40981-024-00742-z

**Published:** 2024-09-17

**Authors:** Junichi Saito, Shino Ichikawa, Reiko Kudo, Kurumi Saito, Masayo Kiyokawa, Tetsuya Kushikata

**Affiliations:** Department of Anesthesiology, Hirosaki University Graduate School of Medicine, 5 Zaifu-Cho, Hirosaki, Aomori 036-8562 Japan

**Keywords:** Regional oxygen saturation, Selective cerebral perfusion, Aortic arch stenosis

## Abstract

**Background:**

We report a pediatric case where bilateral regional oxygen saturation (rSO_2_) measurements were useful in determining the selective cerebral perfusion (SCP) flow rate.

**Case presentation:**

A 9-year-old Japanese boy, 128 cm tall and weighing 25.6 kg, was scheduled for aortic arch reconstruction due to a 90–100 mmHg pressure gradient. Pediatric-sized oximetry sensors were attached to the bilateral forehead area. The rSO_2_ levels were 70–80% on the right and 80–90% on the left during cardiopulmonary bypass. Immediately following deep hypothermic circulatory arrest with the body temperature cooled to 25 °C, SCP was initiated from the right brachiocephalic artery at 10 mL/kg/min. As the rSO_2_ decreased steeply to 43–45% on the right and to 32–38% on the left, the SCP flow was increased to 15 mL/kg/min. The right rSO_2_ increased promptly to 50–60%, but the left rSO_2_ remained at 30–40%. After the SCP flow was increased to 20 mL/kg/min, bilateral rSO_2_ levels of 50–60% were obtained, and the SCP flow rate was maintained. The patient was transferred to the ICU postoperatively and extubated on the second postoperative day with no neurological abnormalities.

**Conclusions:**

Bilateral rSO_2_ measurements are essential even for a pediatric patient undergoing SCP, despite the limited forehead area.

## Background

In pediatric cardiac surgery, the selective cerebral perfusion (SCP) flow rate during circulatory arrest lacks an absolute index, leading to substantial variation in the literature [1]. In both pediatric and adult patients undergoing SCP, it is crucial to ensure a sufficient supply of oxygen to the brain, with various monitoring methods, including regional oxygen saturation (rSO_2_), playing a pivotal role. However, in pediatric patients, rSO_2_ is often measured by applying only one sheet due to the limited area of the frontal region. We herein report a case in which bilateral cerebral rSO_2_ measurements were useful in determining SCP flow rate.

Written informed consent was obtained from the patient’s parent for publication of this case report and accompanying images.

## Case presentation

**Fig. 1:**
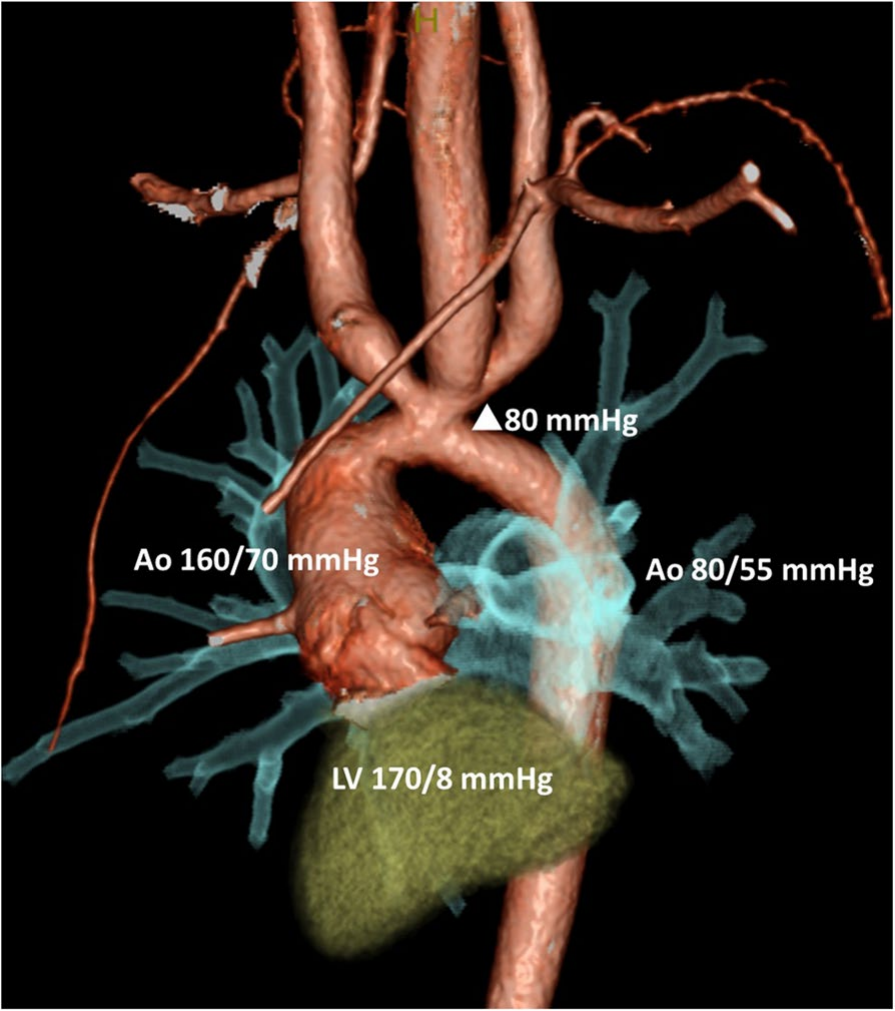
Preoperative images of aortic arch stenosis. Intracardiac and aortic pressure was measured by left ventricular and aortic arch angiography 9 months prior to surgery. Aortic arch stenosis with a pressure gradient about 80 mmHg was found. LV left ventricular, Ao aorta, △ pressure gradient

A 9-year-old Japanese boy, 128 cm tall and weighing 25.6 kg, was scheduled to undergo ascending aorta replacement and aortic arch reconstruction under general anesthesia. He had been diagnosed with interrupted aortic arch type A and ventricular septal defect at birth, and at 18 days of age had undergone aortoplasty and ventricular septal defect closure. Postoperatively, he was found to have aortic arch stenosis and was followed up in our pediatric department. Nine months prior to surgery, transthoracic echocardiography revealed progressive aortic arch stenosis with a maximum pressure gradient of 90–100 mmHg. Preoperative left ventricular angiography and aortic arch angiography indicated pressures of 170/80 mmHg in the left ventricle, 160/70 mmHg in the ascending aorta, and 80/55 mmHg in the descending aorta (Fig. 1). Physical examination revealed a pressure gradient of 30 mmHg between the upper (113/68 [86] mmHg) and lower (84/53 [67] mmHg) extremities.

**Fig. 2:**
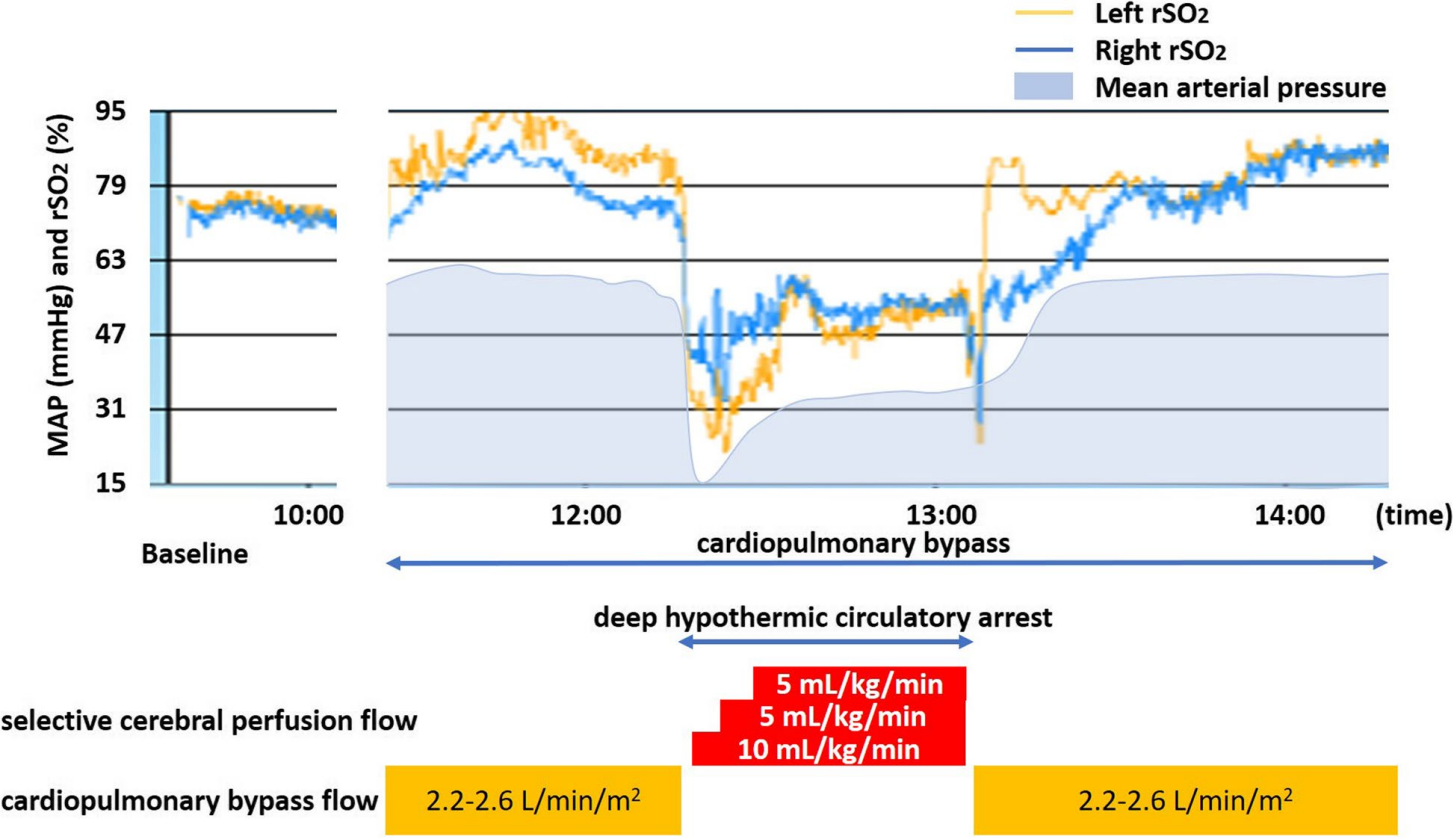
Changes in bilateral cerebral regional oxygen saturation (rSO_2_) and mean arterial pressure. Cerebral bilateral rSO_2_ values changed as the selective cerebral perfusion flow rate increased during deep hypothermic circulatory arrest

An oral dose of 8 mg midazolam was administered as an anesthetic premedication 1 h before the surgery. In addition to the standard American Society of Anesthesiology monitoring, invasive arterial pressure was monitored in the left radial artery, and central venous pressure was assessed via the right internal jugular vein. A bispectral index sensor and pediatric-sized oximetry sensors were placed on the patient’s forehead. A regional oxygen saturation probe (INVOS 5100C™, Medtronic, Minneapolis, MN, USA) was attached to monitor the cerebral rSO_2_ values on both sides. Anesthesia was induced and maintained using remimazolam, midazolam, ketamine, and fentanyl. Following the administration of rocuronium bromide, tracheal intubation was performed without any complications. A blood pressure difference of 40 to 50 mmHg was noted between the left radial artery (130/60 mmHg) and the left dorsal artery (90/55 mmHg). After the induction of anesthesia, the baseline bilateral rSO_2_ values were similar, around 70% (Fig. 2). Once cardiopulmonary bypass (CPB) was established, the body temperature was cooled to 25 °C. The rSO_2_ during CPB was 70–80% on the right side and 80–90% on the left. Antegrade SCP (10 mL/kg/min) via the brachiocephalic artery was initiated immediately after deep hypothermic circulatory arrest (DHCA). As the rSO_2_ dropped sharply to 43–45% on the right and 32–38% on the left, the SCP flow was increased to 15 mL/kg/min. The right rSO_2_ increased promptly to 50–60%, but the left rSO_2_ remained at 30–40%. After the SCP flow was increased to 20 mL/kg/min, bilateral rSO_2_ reached levels of 50–60%, and the SCP flow rate was maintained. Following the ascending aorta replacement and aortic arch reconstruction, the patient was weaned from CPB. The pressure gradient between the left radial and dorsal arteries decreased to 15–20 mmHg at the conclusion of surgery. The patient was admitted to the ICU postoperatively and extubated on the second postoperative day with no neurological abnormalities. The intraoperative data were as follows: duration of DHCA 50 min, duration of CPB 2 h 59 min, duration of surgery 5 h 23 min, duration of anesthesia 6 h 43 min.

## Discussion

Bilateral cerebral rSO_2_ measurements in a patient who had undergone aortic arch reconstruction were useful in determining the SCP flow rate during DHCA. Bilateral cerebral rSO_2_ measurements are necessary because the left side of the brain may exhibit lower rSO_2_ compared to the right side, presumably due to blood flow to the left side during SCP from the brachiocephalic artery occurring via the circle of Willis [2]. Moreover, various anatomic variations and obstructions to left-sided venous drainage may affect the blood flow to the left side of the brain [3]. As mentioned above, a prospective study demonstrated that out of 19 neonatal patients who had undergone Norwood surgery, 9 exhibited sustained differences in rSO_2_ values exceeding 10%, with the left-side values being lower than the right. In some cases, rSO_2_ differences were as large as 30% [3]. Hence, without monitoring the left side of the brain, undetected desaturation in the left cerebral hemisphere could occur.

In the present case, cerebral rSO_2_ values rose in conjunction with the escalation of the SCP flow rate. This rate varies widely in the literature, and the optimal flow rate for effective brain protection during development remains unclear. Although the SCP flow rate was defined as 50 mL/kg/min in the initial description of SCP with the DHCA technique [4], the flow rates varied widely, ranging from 20 to 94 mL/kg/min [1]. To overcome the uncertainties around the SCP flow rate, a suggested approach involved adjusting the flow rate to maintain cerebral rSO_2_ and Doppler flow velocity to within 10% of the baseline values recorded during full-flow CPB [2]. However, thresholds of rSO_2_ associated with central nervous system injury in pediatric cardiac surgery are under investigation and also there was no clear evidence for avoiding the injury during SCP. Some basic and clinical studies revealed that thresholds of near-infrared spectroscopy for cerebral injury were oxygen saturations in the range of 33 to 55% during perioperative period [5–8]. In a piglet model to determine thresholds for neurologic injury, brain tissue lactate accumulation began when cerebral rSO_2_ values decreased to less than 45% [7]. A clinical study also revealed that the development of new or worsened ischemia on postoperative magnetic resonance imaging following the Norwood procedure was associated prolonged low postoperative cerebral rSO_2_ values (rSO_2_ < 45% for > 180 min) [9]. These results suggest that cerebral rSO_2_ values at least 45% should be maintained during SCP. On the contrary, with 14 out of 34 patients exhibiting cerebral rSO_2_ values of 95% [2], there is a potential risk of cerebral hyperperfusion, and brain injury can occur at high cerebral rSO_2_ levels if the guidance for SCP flow relies solely on rSO_2_ measurements. Further studies are needed to elucidate the targeted cerebral rSO_2_ levels during SCP in pediatric patients.

In addition to the evaluation of local tissue ischemia, measurement of rSO_2_ in patients with congenital heart disease has some advantages. The baseline values of rSO_2_ during the induction of anesthesia are clinically important to assess the global cardiopulmonary function of congenital heart disease patients. Previous studies showed that preoperative rSO_2_ values in awake state could be used to predict poor outcomes in patients undergoing congenital heart surgery [10]. Additionally, we have previously reported that baseline rSO_2_ values after induction of anesthesia were associated with several adverse postoperative outcomes [11] and Modestini and colleagues supported our findings; lower baseline rSO_2_ values were associated with a longer ICU and hospital stay, as well as with a longer duration of mechanical ventilation [12]. As shown previously in adult patients undergoing cardiac surgery, low preoperative rSO_2_ values were associated not only with neurologic adverse outcome but also with increased mortality rates [13]. These results suggested that the brain might be an “index organ” reflecting the severity of cardiopulmonary compromise in patients with cardiac disease [14].

This case showed that bilateral cerebral rSO_2_ measurements in a pediatric patient undergoing aortic arch reconstruction were useful in establishing the SCP flow rate during DHCA. Although the limited frontal area of a pediatric patient makes bilateral rSO_2_ measurements difficult, such measurements are indispensable, especially in the context of a pediatric patient undergoing SCP.

## Data Availability

No data available.

## Abbreviations

rSO_2_: Regional oxygen saturation
SCP: Selective cerebral perfusion
CPB: Cardiopulmonary bypass
DHCA: Deep hypothermic circulatory arrest
